# Bridging the Silos: A Comparative Analysis of Implementation Science and Improvement Science

**DOI:** 10.3389/frhs.2021.817750

**Published:** 2022-02-04

**Authors:** Per Nilsen, Johan Thor, Miriam Bender, Jennifer Leeman, Boel Andersson-Gäre, Nick Sevdalis

**Affiliations:** ^1^Division of Society and Health, Department of Health, Medicine and Caring Sciences, Linköping University, Linköping, Sweden; ^2^Jönköping University, Jönköping Academy for Improvement of Health and Welfare, Jönköping, Sweden; ^3^Sue and Bill Gross School of Nursing, University of California, Irvine, Irvine, CA, United States; ^4^School of Nursing, University of North Carolina at Chapel Hill, Chapel Hill, NC, United States; ^5^Health Service & Population Research Department, Centre for Implementation Science, King's College London, London, United Kingdom

**Keywords:** improvement science, quality improvement, implementation science, comparative analysis, context

## Abstract

**Background:**

Implementation science and improvement science have similar goals of improving health care services for better patient and population outcomes, yet historically there has been limited exchange between the two fields. Implementation science was born out of the recognition that research findings and effective practices should be more systematically disseminated and applied in various settings to achieve improved health and welfare of populations. Improvement science has grown out of the wider quality improvement movement, but a fundamental difference between quality improvement and improvement science is that the former generates knowledge for local improvement, whereas the latter is aimed at producing generalizable scientific knowledge.

**Objectives:**

The first objective of this paper is to characterise and contrast implementation science and improvement science. The second objective, building on the first, is to highlight aspects of improvement science that potentially could inform implementation science and vice versa.

**Methods:**

We used a critical literature review approach. Search methods included systematic literature searches in PubMed, CINAHL, and PsycINFO until October 2021; reviewing references in identified articles and books; and the authors' own cross-disciplinary knowledge of key literature.

**Findings:**

The comparative analysis of the fields of implementation science and improvement science centred on six categories: (1) influences; (2) ontology, epistemology and methodology; (3) identified problem; (4) potential solutions; (5) analytical tools; and (6) knowledge production and use. The two fields have different origins and draw mostly on different sources of knowledge, but they have a shared goal of using scientific methods to understand and explain how health care services can be improved for their users. Both describe problems in terms of a gap or chasm between current and optimal care delivery and consider similar strategies to address the problems. Both apply a range of analytical tools to analyse problems and facilitate appropriate solutions.

**Conclusions:**

Implementation science and improvement science have similar endpoints but different starting points and academic perspectives. To bridge the silos between the fields, increased collaboration between implementation and improvement scholars will help to clarify the differences and connections between the science and practice of improvement, to expand scientific application of quality improvement tools, to further address contextual influences on implementation and improvement efforts, and to share and use theory to support strategy development, delivery and evaluation.

## Background

Within health care research and practice, implementation science has emerged as a vital multidisciplinary research field in the wake of the evidence-based medicine/practice movement. Both evidence-based medicine/practice and implementation science address the untapped potential to improve health and welfare of populations through wider and more systematic use of research findings and implementation of empirically supported (“evidence-based”) practices (i.e., clinical interventions, programmes, services, etc.). The ambition is to reduce the research-to-practice gap; that is, the gap between what is known through research to be effective and what is actually practiced or used in various areas of society (1).

In parallel, the field of improvement science developed in the 2000s with similar aims of bridging the gap between ideal and actual care to improve health care quality and, thereby, patient and population outcomes (2, 3). Improvement science has grown out of the wider quality improvement (QI) movement, which entered health care widely in the late 1980s. QI involves process mapping and systems thinking and the use of measurement and tools to assess, plan, execute and evaluate changes to improve patient and population outcomes, system performance and professional development (4, 5). Whereas, the primary aim of QI is to enhance local performance, improvement science is aimed at producing generalizable knowledge within a scientific framework (6–8).

Implementation science and improvement science have similar goals of illuminating how to improve health care services and patient and population outcomes. Glasziou et al. (9) have argued that achieving this ambition requires integrating the “do (the) right things” orientation of implementation science (implementing evidence-based practices) with the “do things right” orientation of improvement science (making sure the practices are done thoroughly, efficiently and reliably). Still, despite a shared ambition, work within the two fields seems to progress largely separately, with limited exchange or cross-reference between researchers and practitioners (10, 11). The QI pioneer Don Berwick [(12), p. 1,182, 1,184] lamented that the evidence-based movement and QI “are often in unhappy tension.”

The overlapping interest of implementation science and improvement science allows for common ground. Several scholars have argued that aligning the two fields could potentially improve treatment and care to benefit patient and population health (13, 14). For example, greater alignment could benefit implementation science scholars' ability to align their work with the terminology and tools such as Root Cause Analysis used by health care practitioners, many of whom have adopted QI approaches to address problems in health care delivery identified by such methods (15). Further, improvement science scholars might benefit from implementation science's growing menu of frameworks and models to categorise determinants of desired changes and provide guidance for implementation processes. Furthermore, research on collaboration between scholars in different fields suggests that bringing researchers with different backgrounds together can speed up research progress and generate new ideas and discoveries in shorter time periods (16, 17).

In this paper, we address the question: why do the two fields function independently and what are the opportunities to bridge the gap? To address this question, our first objective is to characterise and compare implementation science and improvement science as fields of scientific inquiry. Building on this, our second objective is to identify aspects of each field that potentially could inform the other so as to advance both fields. We begin by providing a brief overview of both implementation science and improvement science, using key literature. This is followed by a comparison of key aspects of the two fields, recommendations for how to address key differences, and a discussion of opportunities for cross-fertilisation.

## Methods

We used a critical literature review approach (18), which has been applied in past comparative reviews of related topics, such as knowledge translation (19) and large health care system transformation (20). Search methods included systematic literature searches in PubMed, CINAHL, and PsycINFO until October 2021 (using the search terms “improvement/implementation science,” and “improvement/implementation research”); snowball techniques such as reviewing references in identified articles and books; and the authors' own cross-disciplinary knowledge of key literature. We further searched until October 2021 for relevant content in key disciplinary journals, including *Implementation Science, BMC Health Services Research, BMJ Quality & Safety, BMJ Open Quality, International Journal for Quality in Health Care* and *American Journal of Medical Quality*.

Comparative analysis is a method for comparing two or more topics to identify and analyse similarities and/or differences. The product has the potential to engender a deeper understanding of each topic separately (21). The comparison of implementation science and improvement science used the following categories developed iteratively based on the research question (22):

(1) Influences: origins of the fields and knowledge sources drawn upon

(2) Ontology, epistemology and methodology: characteristics of the research

(3) Problem identification: key problem described in the research

(4) Potential solutions: strategies proposed to address the problem

(5) Analytical tools: theories, models, frameworks and other knowledge products and processes used to analyse, understand and explain problems, and to facilitate appropriate solutions

(6) Knowledge production and use: practice settings in which the research is conducted and users of the knowledge produced

The comparative analysis identified areas of convergence and difference across the fields. From this analysis, we identified and articulated opportunities for cross-fertilisation.

From the self-reflexive perspective on the current disciplinary “boundaries” of the two fields, the authors of the paper are engaged in implementation science and improvement science, with PN primarily involved in implementation science research, JT and BAG primarily involved in improvement science research, and MB, JL, and NS being equally engaged in both fields.

## A Brief History of Implementation Science

The birth of the field of implementation science is usually linked to the emergence of the evidence-based medicine/practice movement in the 1990s. This movement has popularised the notion that the effectiveness of health services depends on consistent application of the best available research findings and empirically supported (“evidence-based”) practices (e.g., preventive, diagnostic or therapeutic interventions, services, programmes, methods, techniques, and routines) to achieve improved health and welfare of populations (23). Spread of the evidence-based medicine/practice movement has been facilitated by developments in information technology, especially electronic databases and the Internet, which have enabled practitioners, policy makers, researchers and others to readily identify, collate, disseminate and access research on a global scale (24). The movement also resonates with many contemporary societal issues and concerns, including the progress of New Public Management, which has highlighted issues of effectiveness, quality, accountability, and transparency (25).

Implementation science is commonly defined as the scientific study of ways to promote the systematic uptake of research findings and other evidence-based practices into routine practice to improve the quality and effectiveness of health services and care (2). The term implementation research is often used interchangeably with implementation science. Other terms in circulation to describe essentially similar research concerning how to put various forms of knowledge to use include knowledge translation, knowledge transfer, knowledge exchange, knowledge integration, and knowledge mobilisation (26, 27).

Although implementation science is a young research field in its own right, research on the challenges associated with how intentions are translated into effective actions to address society's problems has a long history. Many elements of today's implementation science can be traced to research on the spread and adoption of innovations. This research originated in sociology in the early 1900s (28). Everett M. Rogers collated different traditions and presented a conceptual apparatus for the spread and adoption of innovations in his ground-breaking book *Diffusion of Innovations*, which was first published in 1962. The theory originated from his own experience as a farmer and then as an investigator of the spread of agricultural innovations (29).

Today's implementation science is also related to research on policy implementation; that is, the study of “how governments put policies into effect” (30). This research rose to prominence in the 1970s during a period of growing concern about the effectiveness of public policy (31). A policy is a plan or course of action intended to influence and determine decisions and actions (32). This research emerged from the insight that political intentions seldom resulted in the planned changes, which led researchers to investigate what occurred in the policy process and how it affected the results (33).

Implementation science also has many connections with the study of research use (or research utilisation). This research grew out of the social science research field of knowledge utilisation in the 1970s, with Robert F. Rich and Carol H. Weiss being prominent scholars (the term “knowledge utilisation” has also been used as a collective name for all research relating to the use of knowledge). As early as 1975, nursing researchers were building on concepts and theories from knowledge utilisation in research to understand how nurses used research in their clinical practice (34, 35). Many researchers who were active in the field of research use subsequently developed broader research agendas within implementation science.

## A Brief History of Improvement Science

The term “the science of improvement” was first used in a health care context by Langley et al. (36), in the 1996 edition of *The Improvement Guide*. However, approaches used in today's improvement practices date back almost 100 years. An important foundation for QI and, thereby for improvement science, was laid by Walter Shewhart in the 1920s and 1930s. A physicist, engineer, and statistician, he developed statistical methods to reveal key aspects of the quality of industrial processes (37). His work on tools such as control charts to understand and manage process variation and the Plan–Do–Study–Act (PDSA) cycle (originally called simply the Shewhart cycle or the Shewhart learning and improvement cycle) are foundational for QI and core concerns of improvement science. He summarised his work in his 1931 book *Economic Control of Quality of Manufactured Product* (38).

Shewhart worked at Western Electric Company's Hawthorne factory to assist its engineers in improving the quality of telephone hardware. While at Hawthorne, Shewhart mentored both Joseph Juran and William Edwards Deming who went on to champion Shewhart's tools, not least the PDSA cycle (which was also referred to as the Deming cycle). Deming, a statistician, engineer and business consultant, recognised quality as a primary driver for industrial success and subsequently introduced QI tools to post-world War II Japanese industries, particularly the automobile industry (39). Deming's work was summarised in *Out of the Crisis* (40). Joseph Juran, similarly influential, highlighted the idea that quality can be managed through planning, control, and improvement, known as the Juran Trilogy, as outlined in his multiple-edition *Juran's Quality Handbook* (41). The trio of Shewhart, Deming and Juran are often considered the founders of the QI movement (7, 42).

Interest in applying QI approaches to improve health care increased in the 1980s. Concern about wide geographic variations in health care practice led the United States Congress to establish the Agency for Health Care Policy and Research (today the AHRQ, Agency for Healthcare Research and Quality). Twenty-one health care organisations in the United States participated in the National Demonstration Project in Quality Improvement in Health Care (NDP), a 1987 study to investigate the applicability of QI approaches to health care. Many of the organisations showed improved performance and the NDP was extended three more years before evolving into the Institute for Healthcare Improvement (IHI), a not-for-profit organisation that provides leadership and training in health care QI. From its inception, IHI leaders also promoted QI through influential academic writing (43–45).

Attention to quality problems in health care grew in the 1990s, but it was the landmark publication of *To Err is Human* in 1999 by the US Institute of Medicine (today the National Academy of Medicine) that brought quality problems in health care to widespread attention. According to the report, most medical adverse events result primarily from faulty processes and systems, not from isolated failures of individuals (46). This initial report was followed in 2001 by the follow-up report *Crossing the Quality Chasm* (also by the Institute of Medicine), which documented the substantial gap between actual and desired care, and proposed directions for closing it (47). Contemporaneously and also important was the policy report *An Organisation with a Memory*, which was published in 2000 by the Department of Health in the United Kingdom. It reported on the quantity and causes of adverse events in health care organisations and recommended that health care systems learn from safety incidents and act to improve safety (48). These reports provided political, policy, and funding impetus for developing QI into a research endeavour (8, 12, 49). Over the years, organisations such as The Health Foundation in the United Kingdom and the IHI in the United States have supported and disseminated QI and improvement science knowledge widely (50).

The 2000s saw the development of improvement science as a research field based on the recognition that QI needed a scientific knowledge base (51). There is no unified definition of the field and many different definitions have been proposed. Still, some core characteristics can be identified. Definitions typically build on definitions of QI but emphasise the scientific enquiry into health care improvement issues. Hence, these definitions emphasise the systematic and rigorous study of effectiveness; that is, “what works best” (52), when scientifically evaluated, of various QI strategies (5).

A fundamental difference between QI and improvement science is that the former concerns the practical application of knowledge for local improvement, whereas the latter aims at the accumulation of generalizable knowledge. QI generates knowledge for local improvement, and the results are not primarily intended to be generalised beyond the specific setting or population in question. In contrast, the ambition of improvement science is to generate new, scientific, generalizable knowledge (8, 10, 53). Hence, whereas QI focuses on optimising the local benefits of change, improvement science can be said to focus on maximising learning from, and for, improvement (52). The comparative analysis in this paper focuses on improvement science; references to QI are made when addressing aspects of QI that have direct relevance to improvement science.

## Comparative Analysis of Implementation Science and Improvement Science

The comparative analysis of the implementation science and improvement science fields that we conducted centred on six categories, developed iteratively based on the research questions and analysis of the literature. We first describe findings concerning each of the six categories (summarized in Table 1) and then provide recommendations regarding how key differences might be addressed.

**Table 1:** Summary of similarities and differences between implementation science and improvement science across six thematic aspects.

**Aspect**	**Similarities**	**Differences**
Influences	• Both fields ultimately concern practice change • Both fields acknowledge the relevance of psychology for understanding how desired change might be achieved	• The fields have different origins and draw on mostly different sources of knowledge
Ontology, epistemology, and methodology	• The research characteristics of the two fields are largely similar, primarily belonging to the positivist tradition, but with some interpretivist features • Both fields are highly applied in nature, with aspirations to inform practice	
Problem identification	• Both fields describe a gap or chasm between current and optimal care and/or service delivery	• For improvement science, the problem is related to the efficiency, safety, and/or quality of current practice; in implementation science the problem relates to delays in getting effective practices (clinical interventions, programmes, services, etc.) applied systematically in practice
Potential solutions	• The two fields share multiple common strategies, although they use partially different terminology to describe them	• Improvement science posits that quality improvement follows from successful change in the health care system and its processes. Implementation science assumes that implementation of evidence-based practices will reduce or eliminate the problem. The scope of change is broader in improvement science than in implementation science, because a QI initiative is not necessarily limited to application of scientifically supported evidence, but can also involve operations, service quality and efficiency
Analytical tools	• Both fields use analytical tools to analyse problems and to identify possible solutions	• Improvement science uses a range of QI tools, typically adapted for use in health care from the manufacturing industry and management, whereas implementation science emphasises the use of theories, models and frameworks as analytical tools
Knowledge production and use	• Both fields produce knowledge that is both applicable for improved practice and sufficiently generalizable to contribute to scientific knowledge accumulation • Both fields focus on studies in health care but also encompass research carried out in the broader health and welfare services	• Health care practitioners and organisational developers are more likely to have QI and/or improvement science knowledge than implementation science knowledge

### Influences

Implementation science and improvement science ultimately concern practice change. Improving the quality of a health care process or implementing an evidence-based practice implies the need to change aspects of current practice. Hence, describing and analysing change is important in both fields, but they draw on partially different sources of knowledge to achieve this. Improvement science has been informed by its roots in the management and manufacturing fields, and topics and disciplines such as quality, measurement, management, leadership, strategy, and organisational learning (7, 52, 54). Implementation science has different origins, being influenced by medical sciences (and the evidence-based movement), behavioural sciences and social sciences, perhaps most notably the fields of psychology, organisational behaviour, sociology, and political science (33).

An area of commonality in influence across the two fields is the relevance of psychology for understanding how the desired change can be achieved. However, how psychology is utilised in each field is different. Psychology in implementation science has been applied to analyse change and to identify the mechanisms of this change (55). In implementation science, change is usually considered in terms of behaviour change among health care practitioners (56); for example, the extent to which they act in accordance with an evidence-based practice, such as prescribing an antibiotic for a sore throat, adhering to a hygiene recommendation or providing advice on alcohol consumption. Social-cognitive theories from psychology concerning behaviour change are widely used in implementation science (57). These theories focus on individual cognitions (e.g., motivation, attitudes, beliefs, and self-efficacy) as processes that intervene between observable stimuli and responses in specific real-world situations (58).

In improvement science, psychology is part of Deming's System of Profound Knowledge, which is a holistic approach to leadership and management influenced by the theories of pragmatist C.I. Lewis (59). This system identifies the relevance of having knowledge about psychology, variation, the system and having a theory on knowledge to change organisations (42, 45). For Deming, psychology was essential for understanding the human nature of the people in organisations (5). Contributions from psychology that are important to improvement science include knowledge about differences in people and the relevance of both intrinsic and extrinsic motivation underlying behaviours, and how people can be attracted to change (36, 60).

### Ontology, Epistemology, and Methodology

Despite their different backgrounds, the ontology and epistemology of the two fields can be positioned largely within a positivist tradition. Thus, they seek objectivity and use systematic approaches to undertake research. The researcher is assumed to have direct access to the real world, adherent with positivist beliefs concerning the nature of the world (61, 62). It is believed that it is possible to obtain objective knowledge and the research has a focus on generalisation, consistent with positivist notions about the relationship between the researcher and the reality (61, 62). Both fields study the use of strategies to actively influence and change current practice, to reveal assumed cause-and-effect relationships between controllable and editable independent variables and various outcomes (dependent variables).

Reflecting a positivist approach to methodology (63, 64), researchers in the two fields take a controlled and structured approach in conducting research by identifying a clear research topic, adopting a suitable research methodology and implicitly assuming that the role of the researcher is predominantly that of a detached, external observer. Still, interactive and participatory approaches are increasingly emphasised in implementation science (65). Similarly, improvement science researchers acknowledge the importance of pre-understanding and action-oriented approaches to doing research (66, 67). This field has emphasised the importance of accounting for the personal experience, knowledge and intuition of those who are closest to the problem while recognising the need to frame and test these insights scientifically (42). This knowledge is referred to as subject matter knowledge, which is considered to be unique to each practice setting (45).

Both fields have a strong focus on measurement. Implementation science studies involve measurement, with the influence from clinical epidemiology, other medical sciences and the evidence-based movement evident in the preference for systematic reviews to determine the effectiveness of different implementation strategies (68, 69) (even if the strategies might have been applied in very different contexts). Overall, implementation science uses a wide range of research methods, both qualitative and quantitative, to understand and explain the conditions for implementation by identifying determinants, usually divided into barriers and enablers, for successful implementation and to evaluate the effectiveness of various strategies intended to facilitate implementation (1).

The origins of improvement science in industrial manufacturing provide an explanation for the importance of measurement in this field. The concept of “quality” in industrial production was initially bound up with standardisation, using statistics to understand and manage variation, and measurement was therefore recognised early on as critical to the identification and correction of deviations and deficits in the production process (70). Today, improvement science concerns efforts to use measurement for creating feedback loops to promote learning and gauge the impact of changes over time (36, 71).

### Problem Identification

The two fields address a similar problem: that many patients or service users do not receive optimal care or treatment and that efforts to improve on this situation are often challenging, unsystematic, and meet with mixed success. Both fields start from a gap between current and optimal or desired care and treatment. The gap was famously referred to as a “quality chasm” in the US Institute of Medicine (47) report that inspired improvement science and as an “implementation gap” in implementation science (in contrast to an “evidence gap,” which describes lack of evidence on the effectiveness of a practice). However, although the two fields describe a similar problem, the understanding of this problem and how knowledge of the problem can be obtained differ.

In implementation science, the problem is conceptualised as lack or insufficient use of evidence-based practices in current clinical care, which means that practice is not sufficiently informed by empirical research findings (1) and that (often hard won) research insights are left unused. Data on the deviations between current and evidence-based practice and determinants (barriers and facilitators) contributing to those deviations are key to understanding the problem and informing efforts to solve it (72).

Improvement science is premised on the assumption that there is a gap between the way care is being provided and optimal care delivery in relation to safety, efficiency, effectiveness, equity, patient centredness and timeliness, core dimensions of health care quality highlighted by the Institute of Medicine (47). Data on how care is currently being provided are essential to understanding the quality problem (3, 73).

The problem in improvement science can be identified based on clinical audits, quality registries or on local practice-based knowledge (74); for example, unwarranted variation in clinical practice and in patient outcomes, patient complaints about long waiting times in an emergency department, practitioners' experiences with increased incidence of pressure ulcers or performance benchmarking data that indicate avoidably, even unacceptably, high prescription of antibiotics. Hence, the specific problem can be identified by practitioners or researchers in a sort of bottom-up process in local practice settings. In contrast, the problem in implementation science is more likely to be defined by researchers or health care-related authorities, who identify a gap between current practice and a practice that is based on the latest available evidence (1). Thus, problem identification in implementation science studies tends to be based on more of a top-down process.

Scholars in both fields have increasingly engaged in discussions about how to address the influence of context on the gap between current and optimal care and treatment. Researchers in quality improvement have defined context as “everything else that is not the intervention” [(75), p. 605] or as one of three factors influencing the outcomes, the other two being the QI strategies and the QI tools (73, 76) (see below for further details regarding strategies and tools). This is somewhat similar to implementation science, in that the strategy to facilitate the implementation is not considered to be part of the context, instead being viewed as one of five determinant domains: (1) effectiveness of the strategy to facilitate implementation; (2) attributes of the implemented practice (e.g., the perceived complexity and relative advantage of the clinical intervention, programme, service, etc.); (3) features of the adopters (e.g., health care professionals' attitudes, beliefs and motivation concerning the implemented practice); (4) features of the patients or recipients of the implemented practice (e.g., their values and priorities); and (5) contextual influences (72, 77). Hence, implementation science researchers typically view this “everything else” quite broadly in terms of attributes of the implemented practice and features of the adopters and patients.

### Potential Solutions

The two fields propose partially different means to solving the identified problems in current practice. Implementation science starts from the premise that implementation of evidence-based practices will address the problem and contribute to improved patient and population outcomes. Improvement science, meanwhile, examines whether and how QI in health care systems and processes can ameliorate the problems, thus improving clinical practice and patient and population outcomes.

The solutions studied in improvement science are typically called QI strategies, but they are also referred to as QI interventions or QI activities (66, 78). It is common in improvement science to distinguish between QI strategies and QI tools, the latter being instruments and processes used to define and analyse problems (15).

QI and improvement science share many strategies with implementation science. For example, researchers in both fields have referred to the taxonomy developed by the US Agency for Healthcare Research and Quality (AHRQ), consisting of nine types of “off-the-shelf” strategies, including audit and feedback, health care practitioner education, reminder systems, organisational change and financial incentives, regulation and policy (79). Numerous other strategy taxonomies have been developed in implementation science (80), but many of the strategies are essentially the same as in the AHRQ taxonomy. A recent review of both implementation and improvement science studies found they used many common strategies, although terminology differed (13). Hence, even though the problem is defined differently in the two fields, the potential solutions (i.e., strategies) to address the problem overlap markedly.

### Analytical Tools

Both fields apply a range of analytical tools to understand problems, to inform and evaluate solution designs and efforts to facilitate their application in practice. Implementation science places great emphasis on the use of analytical tools in the form of theories, models and frameworks, both to describe and guide actual implementation endeavours (i.e., action models) and to analyse implementation (i.e., determinant frameworks) (72). Some of the theoretical approaches have been developed within the field by researchers from varying backgrounds (including psychology, nursing and sociology), e.g., Consolidated Framework for Implementation Research (77), Normalisation Process Theory (81), Organisational Readiness for Change (82), and the Theoretical Domains Framework (55). Other theories (“classic” theories) have been borrowed from other fields, such as psychology, sociology and organisational behaviour, and tend to be broader in nature (72).

A crucial element of improvement science is the wide range of generic QI tools, inherited from many years of QI work (15), that can be applied to quality and performance problems. Implementation science scholars also borrow some of these tools (13, 14, 80, 83), but they were not developed in this field.

Implementation science studies often investigate health care practitioners' behaviour change as an implementation outcome, emphasising the importance of using theory to understand and explain “what works, for whom and under what circumstances” (55, 84, 85). Similar approaches are entertained in improvement science (86–89). Both fields seek ways to determine cause-and-effect relationships.

### Knowledge Production and Use

The two fields aim to produce knowledge that is applicable and useful in practice while simultaneously sufficiently generalizable for scientific knowledge accumulation. Implementation science studies are conducted in the wider health and welfare services (90, 91). Similarly to implementation science, improvement science research is carried out in health care settings, but studies also go beyond health care to encompass, for example, community-based services, education and social work. The wider QI movement encompasses many other environments, including manufacturing, software development, aviation and the military; that is, sectors that have systematically explored the most effective ways to reduce variability and improve quality (5, 92).

Both fields involve scholars who conduct research on improvement and implementation issues, and practitioners who are actively involved in “doing” QI work and carrying out implementation in real-world settings. However, health care practitioners are currently more likely to be knowledgeable in QI/improvement science than in implementation science (10). Knowledge used in (QI and) improvement science, including information about the numerous QI tools, is increasingly taught in health care practitioners' undergraduate, postgraduate and continuing professional education globally (93, 94). Furthermore, health care practitioners who are employed in organisational or health care development capacities also make use of this knowledge and enable it to be applied in health care practice (11).

In contrast, practitioners in health care and other areas tend not to be knowledgeable about implementation science (10). In fact, a gap has been noted between knowledge about implementation science (e.g., regarding key determinants or the most effective strategies) and the actual use of this knowledge in practice to facilitate implementation endeavours (95). Although there is a proliferation of “evidence-based skills” literature and courses, these tend to focus on how to critically appraise research studies and scientific evidence rather than on how to actually apply it effectively (96). Implementation science researchers have developed action models such as Knowledge-to-Action (97) and Quality Implementation Framework (98) to guide the translation of research into practice, but they are not as hands on or as widely disseminated or used as QI tools. Hence, knowledge produced in implementation science is still predominantly the domain of academia rather than health care practice and management. Paradoxically, there is a risk that valuable research about how to implement research is not being applied effectively in practice.

### Recommendations to Achieve Increased Collaboration Between Implementation Science and Improvement Science

The comparative analysis shows that there are several similarities between the two fields, but there are also numerous differences that would need to be addressed to promote collaboration to allow the fields to learn from each other's approaches, expertise and experiences. The fields have different origins and draw on mostly different sources of knowledge, yet this does not constitute a problem since it can serve to broaden and deepen the understanding of the problem and solutions to produce more useful and indeed deeper knowledge for research and practice. Both fields are inherently multidisciplinary, with scholars who are used to working together with others who might have different backgrounds, including clinicians, health care managers and people with lived experience if illness and care pathways. This suggests that collaboration with scholars and other stakeholders coming from the other field is not a barrier for cross-fertilisation.

The two fields are based on different premises as to what constitutes the problem. The starting point for improvement science is a need or opportunity to improve performance (e.g., efficiency, effectiveness, timeliness, equity), whereas implementation science is based on the recognition that current practice is not sufficiently evidence-based. Rather than viewing these two orientations as conflicting, we recommend that the two fields recognise them as complementary. In practice, problems often include aspects relevant to both perspectives. For example, long waiting times in an emergency department may result from both underuse of evidence-based triage tools and problems concerning care processes. Thus, implementation scientists would benefit from improvement science's process mapping or Root Cause Analysis methods, while improvement science would benefit from a consideration of existing tools that have demonstrated effectiveness in improving triage processes.

The potential solutions to the identified problems also differ between the two fields. The scope for solutions to achieve the desired practice is broader in improvement science than in implementation science simply because QI initiatives are not necessarily limited to application of scientific evidence. Implementation science is usually defined in terms of research on implementing evidence-based practices with convincing empirical support from clinical trials, preferably randomised controlled trials. In practice, however, this definition tends to be applied inconsistently as journals publishing implementation science studies also publish occasional studies involving practices that lack solid empirical support (99, 100). We argue that the focus on practices that are evidence-based limits the ability to assess how important the strength of the evidence is relative to other determinants for implementation success. For example, a highly structured clinical intervention with high efficacy shown in randomised control trials may be harder to implement than an intervention with less evidence, e.g., based on a number of small observational studies. Loosening conceptual restrictions of implementation science to evidence-based practices would introduce the field to the opportunities that are inherent in improvement science, which welcomes any reasonable approach to improvement. Obviously, such a development would considerably reduce the differences between the two fields.

Developing and implementing solutions to identified problems benefits from accounting for local knowledge of relevance for the implementation and/or improvement. In this regard, implementation science scholars could learn from improvement science by considering how local and tacit knowledge (e.g., of frontline health care practitioners) as well as “expertise by experience” (e.g., of service users) is accounted for in improvement efforts when designing tailored implementation strategies. The approach of improvement science coupled with existing knowledge about adaptation in implementation science (101) offers the potential for more tailored, context-sensitive implementation strategies instead of using “off-the-shelf” strategies.

It has been argued that improvement science scholars have achieved a better understanding of the complex concept of context than implementation science scholars (10, 11, 13). Implementation science frameworks that describe determinants of implementation success typically include context as one determinant alongside others, such as attributes of the implemented practice and health care practitioners' beliefs, attitudes, and motivation to change their practice (72). However, the treatment of the context in implementation science, as one of several determinants causally linked to implementation outcomes, implies a fairly reductionist approach to context that often fails to account for the inherent complexity of this concept. Determinant frameworks rarely provide a precise definition or clarify the meaning of the context. Most frameworks define the concept indirectly, in terms of specifying a number of components or dimensions that comprise the context; for example, organisational support, financial resources, culture, and leadership (102). Thus, in many ways, implementation science scholars are still struggling with the concept of context and how to address it in their research. We view this area as an important frontier for both fields to focus their efforts on, particularly in terms of tailoring effective approaches to differing contexts. Research in both fields seems to be heading in precisely this direction. Otherwise, they will remain stuck with the conclusion about the effectiveness of most strategies that “it depends,” without being able to articulate how it does so, or how to adapt to such differences (103).

The two fields use partially different terminology for the solutions developed within each field. However, discussions about the meaning of concepts are not unusual *within* research fields as they evolve over time. For example, both implementation science and improvement science scholars have laboured over how concepts such as context, determinants, frameworks, strategies and interventions should be defined, with considerable within-field inconsistency in the use of many terms (66, 72, 78, 102). Differences in terminology can be a problem, particularly when implementation science scholars engage with practice settings, which are increasingly adopting QI approaches (14). As a result, health care practitioners are learning the language of improvement science. To be successful, implementation science scholars must engage with health care practitioners who are expected to adopt and use their evidence-based practices.

There are also differences with regard to what analytical tools are used in the two fields. We believe implementation science scholars should take a closer look at how improvement science researchers and practitioners use QI tools such as PDSA cycles, Six-Sigma, Root Cause Analysis and Failure Mode, and Effects Analysis (7, 15, 39). These tools can facilitate description and analysis of problems and support the development of relevant solutions. There are still relatively few implementation science studies that use the tools, but interest seems to be increasing, which is encouraging (13, 14).

The importance of using theory to understand the mechanisms of change appears to be more pronounced in implementation science than in improvement science. It has been argued that implementation science can offer valuable insights for improvement science into the how and why of change (11, 104). Improvement science scholars Ramaswamy et al. [(53), p. 15], stress the importance of “unpacking the black box of improvement” to learn what happens during the process of change. Although implementation science now has a strong focus on using theory to understand and explain change, early implementation science was critiqued on the basis of its limited use of theory (105, 106). However, the field has seen wider recognition of the need to establish the theoretical bases of implementation and the strategies used to facilitate implementation (72). A similar development has been advocated in improvement science (88). Increased collaboration between scholars in the two fields could facilitate more emphasis on theory use in improvement science to allow for better understanding and explanation of how and why certain improvements or not are achieved.

The two fields also differ concerning knowledge production and use. We contend that implementation science researchers could learn a great deal from some aspects of improvement science. In many ways, improvement science has a practitioner-friendly “how-to-do-it” orientation that facilitates the use of this knowledge in practice settings. QI/improvement science has been more successful in disseminating knowledge about basic QI principles and QI tools to health care leaders and practitioners, possibly because many accessible QI resources provide practical approaches that health care systems are in need of; that is, standardised ways to improve health care structures and processes that can be taught through training programmes (36, 44). Implementation science seems to have taken note, because recent years have seen a growth in the number of courses and programmes in implementation science directed at both practitioners and researchers, and publications providing more hands on, practical summaries of implementation science approaches; for example, the *Implementation Science Research Development (ImpRes) Tool* (107–112).

Knowledge produced in the course of QI is practice-based and held by practitioners, whereas knowledge generated in implementation science as well as improvement science is research based and therefore predominantly the domain of the academic community. The need to more clearly distinguish between QI and improvement science is a position taken by many improvement science scholars (6, 78, 104, 113, 114). Indeed, scholars have conveyed critique that the field is being held back by people who resist “the suggestion that science should play a more prominent role in improvement” [(104), p. 254] and therefore do not adopt a “more scientific approach to improvement” [(115), p. 83]. We believe such a development would open up more opportunities for collaboration between scholars in the two fields.

## Discussion

This comparative analysis study has sought to characterise implementation science and improvement science, analyse similarities and differences between the two fields, and provide recommendations how to address the differences so that improvement science potentially could inform implementation science and vice versa. At a higher abstraction level, we conclude that the two fields are remarkably similar, with a shared goal of using scientific methods to understand and explain how health care services can be improved for better patient and population outcomes. At lower abstraction levels, our comparative analysis identified some key differences and opportunities for enriching interaction between the fields.

Both fields ultimately concern practice change and describe a problem in terms of a gap or chasm between current and optimal care and treatment. Hence, it is not surprising that numerous scholars in both fields have argued for a merger or increased integration of the two fields. It was not uncommon in the early 2000s for scholars to conduct research in both fields. A 2012 discussion paper in *Implementation Science* (116) conveyed ambitions for a common science concerning research on how to improve health care, but these plans have since been laid to rest. More recently, Koczwara et al. (11), Check et al. (13) and Leeman et al. (14) have called for scholars who are proficient in both fields. A recurrent theme at many of the conferences the authors behind this study have attended is debate concerning whether and how the two fields differ and why there seems to be only limited collaboration—discussions that have prompted this paper.

Despite such calls for integration between implementation science and improvement science, they have not yet found adequate common ground. Why? After all, both fields ultimately are concerned with carrying out structured, rigorous and systematic scientific processes to build scientific knowledge to inform improvement of health and health care. In light of this study, we take the view that part of the continued separation between the two fields can be attributed to a failure to distinguish between QI and improvement science, with impressions of improvement science being influenced by views of QI as not being scientific (104, 117) and relying too much on “intuition and anecdotal accounts” [(15), p. 138]. Conversely, the challenges of applying implementation science in practice may perpetuate this separation.

We believe collaboration between the two fields will be more likely as improvement science matures as a scientific endeavour that is distinct from QI (even though QI tools might be used). Increased use of QI tools in implementation science and practice may also contribute to increased interactions between the two fields. Ultimately, integration will depend on a genuine interest among scholars (and indeed practitioners) to learn about each other's fields and collaboration to create favourable conditions for synergies. A comparative analysis like this is bound to identify many aspects that differ, yet the two fields have the same ambitions to produce scientific knowledge for improved patient and population outcomes; an inclusive approach to evidence-informed improvement through cross-field collaboration can achieve these ambitions more quickly and effectively.

## Conclusions

Our comparative analysis identified both similarities and differences between implementation science and improvement science. The two fields have disparate origins and draw on mostly different sources of knowledge but have a shared goal of using scientific methods to understand and explain how health care services can be improved for better patient and population outcomes. The two fields describe a problem in terms of a gap or chasm between current and optimal care and treatment, and use similar strategies to address the problems. Both fields apply a range of analytical tools to understand problems and inform effective solutions, but implementation science is more focused on using tools (theories, models, frameworks) to disentangle the mechanisms of change to explain the how and why of practice change.

Increased collaboration between scholars (and practitioners) in the two fields, clarifying the differences between the science of improvement and its practice-oriented predecessor, QI, expanded scientific application and evaluation of QI tools, advanced analysis of ways to manage contextual influences on implementation and improvement efforts, and more coherent and shared use of theory to support strategy development, delivery and evaluation can all help move both fields forward and bridge the silos between them.

## Data Availability Statement

The original contributions presented in the study are included in the article/supplementary material, further inquiries can be directed to the corresponding author/s.

## Author Contributions

All authors contributed to the conception, design, analysis and interpretation of data, and writing the manuscript.

## Funding

NS was supported by the National Institute for Health Research (NIHR) Applied Research Collaboration (ARC) South London at King's College Hospital NHS Foundation Trust. NS was a member of King's Improvement Science, which offers co-funding to the NIHR ARC South London and was funded by King's Health Partners (Guy's and St Thomas' NHS Foundation Trust, King's College Hospital NHS Foundation Trust, King's College London and South London and Maudsley NHS Foundation Trust), and Guy's and St Thomas' Foundation. NS was further supported by the ASPIRES research programme (Antibiotic Use Across Surgical Pathways–Investigating, Redesigning and Evaluating Systems), funded by the Economic and Social Research Council. NS was further funded by the NIHR Global Health Research Unit on Health System Strengthening in Sub-Saharan Africa, King's College London (GHRU 16/136/54) using UK aid from the UK Government to support global health research.

## Author Disclaimer

The views expressed are those of the authors and not necessarily those of the NHS, the NIHR, or the Department of Health and Social Care.

## Conflict of Interest

NS is the director of London Safety and Training Solutions Ltd, which offers training in patient safety, implementation solutions and human factors to health care organisations and the pharmaceutical industry. The remaining authors declare that the research was conducted in the absence of any commercial or financial relationships that could be construed as a potential conflict of interest.

## Publisher's Note

All claims expressed in this article are solely those of the authors and do not necessarily represent those of their affiliated organizations, or those of the publisher, the editors and the reviewers. Any product that may be evaluated in this article, or claim that may be made by its manufacturer, is not guaranteed or endorsed by the publisher.

## References

[1] Bauer MS Damschroder L Hagedorn H Smith J Kilbourne AM. An introduction to implementation science for the non-specialist. BMC Psychol. (2015) 3:32. 10.1186/s40359-015-0089-9 26376626 PMC4573926

[2] Grol R Wensing M Eccles M. Improving Patient Care: The Implementation of Change in Clinical Practice. Edinburgh: Elsevier (2005).

[3] Ting HH Shojania KG Montori VM Bradley EH. Quality improvement: science and action. Circulation. (2009) 119:1962–74. 10.1161/CIRCULATIONAHA.108.768895 19364989

[4] Batalden PB Davidoff F. What is “quality improvement” and how can it transform healthcare? Qual Saf Health Care. (2007) 16:2–3. 10.1136/qshc.2006.022046 17301192 PMC2464920

[5] Wagstaff DT Bedford J Moonesinghe SR. Improvement science in anaesthesia. Curr Anesthesiol Rep. (2017) 7:432–9. 10.1007/s40140-017-0234-5 29200976 PMC5696442

[6] Reinhardt AC Ray LN. Differentiating quality improvement from research. Appl. Nurs. Res. (2003) 16:2–8. 10.1053/apnr.2003.59000 12624857

[7] Varkey P Reller K Resar RK. Basics of quality improvement in health care. Mayo Clin Proc. (2007) 82:735–9. 10.1016/S0025-6196(11)61194-4 17550754

[8] Flynn R Scott SD Rotter T Hartfield D. The potential for nurses to contribute to and lead improvement science in health care. J Adv Nurs. (2016) 73:97–107. 10.1111/jan.13164 27682155

[9] Glasziou P Ogrinc G Goodman S. Can evidence-based medicine and clinical quality improvement learn from each other? BMJ Qual Saf. (2011) 20:i13–7. 10.1136/bmjqs.2010.046524 21450763 PMC3066698

[10] Övretveit J Mittman B Rubenstein L Ganz DA. Using implementation tools to design and conduct quality improvement projects for faster and more effective implementation. Int J Health Care Qual Assur. (2017) 30:1–17. 10.1108/IJHCQA-01-2017-0019 28958203

[11] Koczwara B Stover AM Davies L Davis MM Fleisher L Ramanadhan S. Harnessing the synergy between improvement science and implementation science in cancer: a call to action. J Oncol Pract. (2018) 14:335–40. 10.1200/JOP.17.00083 29750579 PMC6075851

[12] Berwick BM. The science of improvement. JAMA. (2008) 299:1182–4. 10.1001/jama.299.10.1182 18334694

[13] Check DK Zullig LL Davis MM Davies L Chambers D Fleisher L. Improvement science and implementation science in cancer care: identifying areas of synergy and opportunities for further integration. J Gen Intern Med. (2020) 36:186–95. 10.1007/s11606-020-06138-w 32869193 PMC7859137

[14] Leeman J Rochweder C Lee M Brenner A Dwyer A Ko LK. Aligning implementation science with improvement practice: a call to action. Implement Sci. Commun. (2021) 2:99. 10.1186/s43058-021-00201-1 34496978 PMC8424169

[15] Hughes RG. Tools and strategies for quality improvement and patient safety. In: Hughes RG editors. Patient Safety and Quality: An Evidence-Based Handbook for Nurses. Rockville, MD: Agency for Healthcare Research and Quality (2008). 21328752

[16] Melin G. Pragmatism and self-organization: research collaboration on the individual level. Res Policy. (2000) 29:31–40. 10.1016/S0048-7333(99)00031-1

[17] Lee S Bozeman B. The impact of research collaboration on scientific productivity. Soc Stud Sci. (2005) 35:673–702. 10.1177/030631270505235918796149

[18] Jesson J Lacey F. How to do (or not to do) a critical literature review. Pharm Educ. (2006) 6:139–48. 10.1080/15602210600616218

[19] Greenhalgh T Wieringa S. Is it time to drop the “knowledge translation” metaphor? A critical literature review. J R Soc Med. (2011) 104:501–9. 10.1258/jrsm.2011.110285 22179293 PMC3241522

[20] Best A Greenhalgh T Lewis S Saul JE Carroll S Bitz J. Large-system transformation in health care: a realist review. Milbank Q. (2012) 90:421–56. 10.1111/j.1468-0009.2012.00670.x 22985277 PMC3479379

[21] Ragin CC. The Comparative Method: Moving Beyond Qualitative and Quantitative Strategies. Berkeley, CA: University of California Press (2014). 10.1525/9780520957350

[22] Petticrew M Roberts H. Systematic Reviews in the Social Sciences: A Practical Guide. Oxford: Blackwell (2008)

[23] Colditz GA. The promise and challenges of dissemination and implementation research. In: Brownson RC Colditz GA Proctor EK editors. Dissemination and Implementation Research in Health. New York, NY: Oxford University Press (2012). p. 3–22. 10.1093/acprof:oso/9780199751877.003.0001

[24] Trinder L Reynolds S. Evidence-Based Practice – A Critical Appraisal. Oxford: Blackwell Science (2000). 10.1002/9780470699003

[25] Hill M. The Public Policy Process. 5th edn. Harlow, ND: Pearson Education (2009).

[26] Estabrooks CA Thompson DS Lovely JJE Hofmeyer A. A guide to knowledge translation theory. J Contin Educ Health Prof. (2006) 26:25–36. 10.1002/chp.48 16557511

[27] Rabin BA Brownson RC. Developing the terminology for dissemination and implementation research. In: Brownson RC. Colditz GA, Proctor EK. Dissemination and Implementation Research in Health. Oxford: Oxford University Press (2012). p. 23–54. 10.1093/acprof:oso/9780199751877.003.0002

[28] Dearing JW Kee KF. Historical roots of dissemination and implementation science. In: Brownson RC Colditz GA Proctor EK editors. Dissemination and Implementation Research in Health. Oxford: Oxford University Press (2012). p. 55–71. 10.1093/acprof:oso/9780199751877.003.0003

[29] Rogers E. Diffusion of Innovations. 5th edn. New York, NY: Simon and Schuster (2003).

[30] Howlett M Ramesh M. Studying Public Policy: Policy Cycles and Policy Subsystems. Oxford: Oxford University Press (2003).

[31] O'Toole LJ Jr. Research on policy implementation: assessment and prospects. J. Public Admin Res Theory. (2000) 10:263–88. 10.1093/oxfordjournals.jpart.a024270

[32] Hill M Hupe P. Implementing Public Policy. 2nd edn. Los Angeles, CA: Sage Publications (2009).

[33] Nilsen P Ståhl C Roback K Cairney P. Never the twain shall meet? A comparison of implementation science and policy implementation research. Implement Sci. (2013) 8:63. 10.1186/1748-5908-8-63 23758952 PMC3686664

[34] Funk SG Tornquist EM Champagne MT. Barriers and facilitators of research utilization. Nurs Clin North Am. (1995) 30:395–407. 7567566

[35] Stetler CB. Stetler model. In: Rycroft-Malone J Bucknall T editors. Models and Frameworks for Implementing Evidence-Based Practice. Chichester: Wiley-Blackwell (2010) 51–82.

[36] Langley GJ Moen RD Nolan KM Nolan TW Norman CL Provost LP. The Improvement Guide: A Practical Approach to Enhancing Organizational Performance. San Francisco, CA: Jossey-Bass (1996).

[37] Berwick DM. Controlling variation in health care: a consultation from Walter Shewhart. Med Care. (1991) 29:1212–25. 10.1097/00005650-199112000-00004 1745079

[38] Shewart WA. Economic Control of Quality of Manufactured Product (Reprint). New York, NY: John Wiley (2015).

[39] Santore MT Islam S. Quality improvement 101 for surgeons: navigating the alphabet soup. Semin Pediatr Surg. (2015) 24:267–70. 10.1053/j.sempedsurg.2015.08.001 26653158

[40] Deming WE. Out of the Crisis. Cambridge, MA: Massachusetts Institute of Technology, Center for Advanced Engineering Study (1986). p. 419–25.

[41] Juran JM Feo DJ. A Juran's Quality Handbook: The Complete Guide to Performance Excellence, 6th edn. New York, NY: McGraw-Hill (2010).

[42] Perla RJ Provost LP Parry GJ. Seven propositions of the science of improvement: exploring foundations. Q Manage Health Care. (2013) 22:170–86. 10.1097/QMH.0b013e31829a6a15 23807130

[43] Berwick DM. Continuous improvement as an ideal in health care. N Engl J Med. (1989) 320:53–6. 10.1056/NEJM198901053200110 2909878

[44] Berwick DM. A primer on leading the improvement of systems. BMJ. (1996) 312:619–22. 10.1136/bmj.312.7031.619 8595340 PMC2350403

[45] Batalden PB Stoltz PK. A framework for the continual improvement of health care: building and applying professional and improvement knowledge to test changes in daily work. Jt Comm J Qual Improv. (1993) 19:424–47; discussion 48–52. 10.1016/S1070-3241(16)30025-6 8252125

[46] Kohn LT Corrigan JM Donaldson MS. To Err Is Human: Building a Safer Health System. A Report of the Committee on Quality of Health Care in America, Institute of Medicine. Washington, DC: National Academies Press (2000). 25077248

[47] Institute of Medicine. Crossing the Quality Chasm: A New Health System for the 21st Century. Washington, DC: Institute of Medicine (2001). 11549951

[48] Department of Health. An Organization with a Memory. London: Department of Health (2000).

[49] Alexander JA Hearld LR. The science of quality improvement implementation. Med Care. (2011) 49:S6–20. 10.1097/MLR.0b013e3181e1709c 20829724

[50] Siriwardena AN. Increasing the impact of quality improvement science: learning from the past and changing the future. Qual Prim Care. (2011) 19:1–2. 21703105

[51] Grol R Baker R Moss F. Quality improvement research: understanding the science of change in health care. Qual Saf Health Care. (2002) 11:110–1. 10.1136/qhc.11.2.110 12448794 PMC1743590

[52] The Health Foundation. Health Foundation Evidence Scan: Improvement Science. London: The Health Foundation (2011). p. 1–21.

[53] Ramaswamy R Reed J Livesley N Boguslavsky V Garcia-Elorrio E Sax S. Unpacking the black box of improvement. Int J Qual Health Care. (2018) 30:15–9. 10.1093/intqhc/mzy009 29462325 PMC5909642

[54] Peden CJ Rooney KD. The science of improvement as it relates to quality and safety in the ICU. J Intensive Care Soc. (2009) 10:260–5. 10.1177/175114370901000409

[55] Michie S Atkins L West R. The Behaviour Change Wheel: A Guide to Designing Interventions. London: Silverback Publishing (2014).

[56] Cane J O'Connor D Michie S. Validation of the theoretical domains framework for use in behaviour change and implementation research. Implement Sci. (2012) 7:37. 10.1186/1748-5908-7-37 22530986 PMC3483008

[57] Nilsen P Roback K Broström A Ellström PE. Creatures of habit: accounting for the role of habit in implementation research on clinical behaviour change. Implement Sci. (2012) 7:53. 10.1186/1748-5908-7-53 22682656 PMC3464791

[58] Fiske ST Taylor SE. Social Cognition. Los Angeles, CA: Sage (2013). 10.4135/9781446286395

[59] Mauléon Mauléon C Bergman B. Exploring the epistemological origins of Shewhart's and Deming's theory of quality: influences from C.I. Lewis' conceptualistic pragmatism. Int J Qual Serv Sci. (2009) 1:160–71. 10.1108/17566690910971436

[60] Health Quality Ontario. Quality Improvement Guide. Health Quality Ontario (2012).

[61] Audi R. Epistemology: A Contemporary Introduction to the Theory of Knowledge. London: Routledge Press (2003).

[62] Chalmers AF. What Is This Thing Called Science? 4th Edn. Buckingham: Open University Press (2004).

[63] Neuman LW. Social Research Methods: Qualitative and Quantitative Approaches. 4th edn. Boston, MA: Allyn and Bacon (2000).

[64] Carson D Gilmore A Perry C Gronhaug K. Qualitative Marketing Research. London: Sage (2001). 10.4135/9781849209625

[65] Ramanadhan S Davis MM Armstrong R Baquero B Ko LK Leng JC. Participatory implementation science to increase the impact of evidence-based cancer prevention and control. Cancer Causes Control. (2018) 29:363–9. 10.1007/s10552-018-1008-1 29417296 PMC5858707

[66] Lynn J Baily MA Bottrell M Jennings B Levine RJ Davidoff F. The ethics of using quality improvement methods in health care. Ann Intern Med. (2007) 146:666–73. 10.7326/0003-4819-146-9-200705010-00155 17438310

[67] Perla RJ Parry GJ. The epistemology of quality improvement: it's all Greek. BMJ Qual Saf. (2011) 20:i24–7. 10.1136/bmjqs.2010.046557 21450765 PMC3066836

[68] Bero LA Grilli R Grimshaw JM Harvey E Oxman AD Thomson MA. Closing the gap between research and practice: an overview of systematic reviews of interventions to promote the implementation of research findings. BMJ. (1998) 317:465–8. 10.1136/bmj.317.7156.465 9703533 PMC1113716

[69] Lau R Stevenson F Ong BN Dziedzic K Treweek S Eldridge S. Achieving change in primary care: effectiveness of strategies for improving implementation of complex interventions: systematic review of reviews. BMJ Open. (2015) 5:e009993. 10.1136/bmjopen-2015-009993 26700290 PMC4691771

[70] Junghans T. “Don't mind the gap!” Reflections on improvement science as a paradigm. Health Care Anal. (2018) 26:124–39. 10.1007/s10728-017-0353-7 29147898 PMC5899994

[71] Thor J Lundberg J Ask J Olsson J Carli C Härenstam KP. Application of statistical process control in healthcare improvement: systematic review. Qual Saf Health Care. (2007) 16:387–99. 10.1136/qshc.2006.022194 17913782 PMC2464970

[72] Nilsen P. Making sense of implementation theories, models and frameworks. Implement Sci. (2015) 10:53. 10.1186/s13012-015-0242-0 25895742 PMC4406164

[73] Portela MC Lima SML Martins M Travassos C. Improvement science: conceptual and theoretical foundations for its application to healthcare quality improvement. Cadernos De Saúde Pública. (2016) 32:111. 10.1590/0102-311X00105815 27828676

[74] Rubenstein LV Hempel S Farmer MM Asch SM Yano EM Dougherty D. Finding order in heterogeneity: types of quality-improvement intervention publications. Qual Saf Health Care. (2008) 17:403–8. 10.1136/qshc.2008.028423 19064654

[75] Övretveit JC Shekelle PG Dy SM McDonald KM Hempel S Pronovost . How does context affect interventions to improve patient safety? An assessment of evidence from studies of five patient safety practices and proposals for research. BMJ Qual Saf. (2011) 20:604–10. 10.1136/bmjqs.2010.047035 21493589

[76] Kaplan CH Provost LP Froehle CM Margolis PA. The model for understanding success in quality (MUSIQ): building a theory of context in healthcare quality improvement. BMJ Qual Saf. (2012) 21:13–20. 10.1136/bmjqs-2011-000010 21835762

[77] Damschroder LJ Aron DC Keith RE Kirsh SR Alexander JA Lowery JC. Fostering implementation of health services research findings into practice: a consolidated framework for advancing implementation science. Implement Sci. (2009) 4:50. 10.1186/1748-5908-4-50 19664226 PMC2736161

[78] Shojania KG Grimshaw JM. Evidence-based quality improvement: the state of the science. Health Affairs. (2005) 24:138–50. 10.1377/hlthaff.24.1.138 15647225

[79] Shojania KG McDonald KM Wachter RM Owens DK. Closing the quality gap: a critical analysis of quality improvement strategies. Volume 1—Series overview and methodology. Technical Review 9 (Contract no. 290-02-0017 to the Stanford University–UCSF Evidence-Based Practices Center). AHRQ Publication No. 04-0051-1. Rockville, MD: Agency for Healthcare Research and Quality (2004). 20734525

[80] Forman-Hoffman VL Middleton JC. McKeeman JL Stambaugh LF Christian RB Gaynes BN . Quality improvement, implementation, and dissemination strategies to improve mental health care for children and adolescents: a systematic review. Implement Sci. (2017) 12:93. 10.1186/s13012-017-0626-4 28738821 PMC5525230

[81] May C Mair FS Finch T. MacFarlane A Dowick C Treweek S . Development of a theory of implementation and integration: normalization process theory. Implement Sci. (2009) 4:29. 10.1186/1748-5908-4-29 19460163 PMC2693517

[82] Weiner BJ. A theory of organizational readiness to change. Implement Sci. (2009) 4:67. 10.1186/1748-5908-4-67 19840381 PMC2770024

[83] Balasubramanian BA Cohen DJ Davis MM Gunn R Dickinson LM Miller WL. Learning evaluation: blending quality improvement and implementation research methods to study healthcare innovations. Implement Sci. (2015) 10:31. 10.1186/s13012-015-0219-z 25889831 PMC4357215

[84] Michie S Johnston M Abraham C Lawton R Parker D Walker A. Psychological theory group. Making psychological theory useful for implementing evidence based practice: a consensus approach. Qual Saf Health Care. (2005) 14:26–33. 10.1136/qshc.2004.011155 15692000 PMC1743963

[85] Grimshaw JM Eccles MP Lavis JN Hill SJ Squires JE. Knowledge translation of research findings. Implement Sci. (2012) 7:50. 10.1186/1748-5908-7-50 22651257 PMC3462671

[86] Walshe K. Understanding what works – and why – in quality improvement: the need for theory-driven evaluation. Int J Qual Health Care. (2007) 19:57–9. 10.1093/intqhc/mzm004 17337518

[87] Reed JE McNicholas C Woodcock T Issen L Bell D. Designing quality improvement initiatives: the action effect method, a structured approach to identifying and articulating programme theory. BMJ Qual Saf. (2014) 23:1040–8. 10.1136/bmjqs-2014-003103 25319412

[88] Reed JE Howe C Doyle C Bell D. Simple rules for evidence translation in complex systems: a qualitative study. BMC Med. (2018) 16:92. 10.1186/s12916-018-1076-9 29921274 PMC6009041

[89] Davidoff F Dixon-Woods M Leviton L Michie S. Demystifying theory and its use in improvement. BMJ Qual Saf. (2015) 24:228–38. 10.1136/bmjqs-2014-003627 25616279 PMC4345989

[90] Brekke JS Ell K Palinkas LA. Translational science at the national institute of mental health: can social work take its rightful place? Res Soc Work Pract. (2007) 17:123–33. 10.1177/1049731506293693

[91] Cook BG Odom SL. Evidence-based practices and implementation science in special education. Except Child. (2013) 79:135–44. 10.1177/0014402913079002021

[92] Lewis S. Qualitative inquiry and research design: choosing among five approaches. Health Promot Pract. (2015) 16:473–5. 10.1177/1524839915580941

[93] Wong BM Etchells EE Kuper A Levinson W Shojania KG. Teaching quality improvement and patient safety to trainees: a systematic review. Acad Med. (2010) 85:1425–39. 10.1097/ACM.0b013e3181e2d0c6 20543652

[94] Armstrong G Headrick L Madigosky W Ogrinc G. Designing education to improve care. Jt Comm J Qual Patient Saf. (2012) 38:5–14. 10.1016/S1553-7250(12)38002-1 22324186

[95] Westerlund A. The Role of Implementation Science in Healthcare Improvement Efforts. (Medical dissertation), Sweden: Umeå University (2018).

[96] Nilsen P Neher M Ellström PE Gardner B. Implementation of evidence-based practice from a learning perspective. Worldviews Evid Based Nurs. (2017) 14:192–9. 10.1111/wvn.12212 28281328

[97] Graham ID Logan J Harrison MB Straus SE Tetroe J Caswell W. Lost in knowledge translation: time for a map? J Contin Educ Health Prof. (2006) 26:13. 10.1002/chp.47 16557505

[98] Meyers DC Durlak JA Wandersman A. The quality implementation framework: a synthesis of critical steps in the implementation process. Am J Community Psychol. (2012) 50:462–80. 10.1007/s10464-012-9522-x 22644083

[99] Bast LS Due P Bendtsen P Ringgard L Wohllebe L Damsgaard MT. High impact of implementation on school-based smoking prevention: the X:IT study – a cluster-randomized smoking prevention trial. Implement Sci. (2016) 11:125. 10.1186/s13012-016-0490-7 27640187 PMC5027074

[100] Hegger I Marks LK Janssen SWJ Schuit AJ Keijsers JFM van Oers HAM. Research for policy (R4P): development of a reflection tool for researchers to improve knowledge utilization. Implement Sci. (2016) 11:133. 10.1186/s13012-016-0496-1 27716245 PMC5045649

[101] Chambers DA Norton WE. The adaptome: advancing the science of intervention adaptation. Am J Prev Med. (2016) 51 (4 Suppl. 2):S124–31. 10.1016/j.amepre.2016.05.011 27371105 PMC5030159

[102] Nilsen P Bernhardsson S. Context matters in implementation science: a scoping review of determinant frameworks that describe contextual influences on implementation outcomes. BMC Health Serv Res. (2019) 19:189. 10.1186/s12913-019-4015-3 30909897 PMC6432749

[103] Pawson R Greenhalgh J Brennan C Glidewell E. Do reviews of healthcare interventions teach us how to improve healthcare systems? Soc Sci Med. (2014) 114:129–37. 10.1016/j.socscimed.2014.05.032 24929647

[104] Marshall M Pronovost P Dixon-Woods M. Promotion of improvement as a science. Lancet. (2013) 381:419–21. 10.1016/S0140-6736(12)61850-9 23374480

[105] Eccles M Grimshaw J Walker A Johnston M Pitts N. Changing the behavior of healthcare professionals: the use of theory in promoting the uptake of research findings. J Clin Epidemiol. (2005) 58:107–12. 10.1016/j.jclinepi.2004.09.002 15680740

[106] Sales A Smith J Curran G Kochevar L. Models, strategies, and tools. J Gen Intern Med. (2006) 21:S43–9. 10.1111/j.1525-1497.2006.00362.x 16637960 PMC2557135

[107] Carlfjord S Roback K Nilsen P. Five years' experience of an annual course on implementation science: an evaluation among course participants. Implement Sci. (2017) 12:101. 10.1186/s13012-017-0618-4 28768546 PMC5541724

[108] Chambers DA Proctor EK Brownson RC Straus SE. Mapping training needs for dissemination and implementation research: lessons from a synthesis of existing D&I research training programs. Transl Behav Med. (2017) 7:593–601. 10.1007/s13142-016-0399-3 27030472 PMC5645270

[109] Proctor EK Chambers DA. Training in dissemination and implementation research: a field-wide perspective. Transl Behav Med. (2017) 7:624–35. 10.1007/s13142-016-0406-8 27142266 PMC5645271

[110] Tabak RG Padek MM Kerner JF Stange KC Proctor EK Dobbins MJ. Dissemination and implementation science training needs: insights from practitioners and researchers. Am J Prev Med. (2017) 52:S322–9. 10.1016/j.amepre.2016.10.005 28215389 PMC5321656

[111] Ginossar T Heckman CJ Cragun D Quintiliani LM Proctor EK Chambers DA. Bridging the chasm: challenges, opportunities, and resources for integrating a dissemination and implementation science curriculum into medical education. J Med Educ Curric Dev. (2018) 5:2382120518761875. 10.1177/2382120518761875 29707648 PMC5892792

[112] King's College London. Implementation Science Research Development (ImpRes) Tool: A Practical Guide to Using the ImpRes Tool. London: King's College London (2018).

[113] Lynn J. When does quality improvement count as research? Human subject protection and theories of knowledge. Qual Saf Health Care. (2004) 13:67–70. 10.1136/qshc.2002.002436 14757803 PMC1758070

[114] Mold JW Peterson KA. Primary care practice-based research networks: working at the interface between research and quality improvement. Ann Fam Med. (2005) 3:S12. 10.1370/afm.303 15928213 PMC1466954

[115] Skela-Savič B Macrae R Lillo-Crespo M Rooney KD. The development of a consensus definition for healthcare improvement science (HIS) in seven European countries: a consensus methods approach. Slovenian J Public Health. (2017) 56:82–90. 10.1515/sjph-2017-0011 28289467 PMC5329771

[116] Wensing M Grimshaw JM Eccles MP. Does the world need a scientific society for research on how to improve healthcare? Implement Sci. (2012) 7:10. 10.1186/1748-5908-7-10 22376988 PMC3292444

[117] Crisp H. Building the field of improvement science. Lancet. (2015) 385:S4–5. 10.1016/S0140-6736(15)60320-8 25726449

